# Monitoring the Modifications of the Vitreous Humor Metabolite Profile after Death: An Animal Model

**DOI:** 10.1155/2015/627201

**Published:** 2015-01-22

**Authors:** Maria Francesca Rosa, Paola Scano, Antonio Noto, Matteo Nioi, Roberta Sanna, Francesco Paribello, Fabio De-Giorgio, Emanuela Locci, Ernesto d'Aloja

**Affiliations:** ^1^Department of Public Health, Clinical and Molecular Medicine, Forensic Science Unit, University of Cagliari, SS 554 Bivio per Sestu, 09042 Monserrato, Italy; ^2^Department of Chemical and Geological Sciences, University of Cagliari, SS 554 Bivio per Sestu, 09042 Monserrato, Italy; ^3^Department of Surgery, University of Cagliari, SS 554 Bivio per Sestu, 09042 Monserrato, Italy; ^4^Public Health Institute, Forensic Science Unit, Catholic University, Largo Francesco Vito 1, 00165 Rome, Italy

## Abstract

We applied a metabolomic approach to monitor the modifications occurring in goat vitreous humor (VH) metabolite composition at different times (0, 6, 12, 18, and 24 hours) after death. The ^1^H-NMR analysis of the VH samples was performed for the simultaneous determination of several metabolites (i.e., the metabolite profile) representative of the VH *status* at different times. Spectral data were analyzed by Principal Component Analysis (PCA) and by Orthogonal Projection to Latent Structures (OPLS) regression technique. PCA and OPLS suggested that different spectral regions were involved in time-related changes. The major time-related compositional changes, here detected, were the increase of lactate, hypoxanthine, alanine, total glutathione, choline/phosphocholine, creatine, and *myo*-inositol and the decrease of glucose and 3-hydroxybutyrate. We attempted a speculative interpretation of the biological mechanisms underlying these changes. These results show that multivariate statistical approach, based on ^1^H NMR metabolite profiling, is a powerful tool for detecting ongoing differences in VH composition and may be applied to investigate several physiological and pathological conditions.

## 1. Introduction 

The vitreous humor (VH) is a highly hydrated tissue with a water content of between 98% and 99.7%. The VH is, in essence, a dilute extracellular matrix. It contains a low number of macrophage-like cells called hyalocytes, fibrillar proteins (mainly collagens), charged carbohydrates, and particularly glycosaminoglycans (GAGs) (mainly hyaluronan). VH is surrounded by, and attached to, the retina, the* pars plana*, and the lens [1]. It has several physiological functions, among which to provide a conduit for the metabolic requirements of the lens and to exclude cells and large macromolecules from the vitreous cavity in order to maintain transparency [2, 3]. Communications between the VH and the retina are granted by the presence of the blood-retinal barrier (BRB), consisting of an inner and an outer component and playing a fundamental role in the microenvironment of the retina and retinal neurons. The BRB is thought to play an essential role in supplying nutrients to the neuroretina in order to maintain visual functions and in preventing leakage of macromolecules and other potentially harmful agents into the retina. In the eye, influx-efflux of compounds, between the ocular vascular bed and retinal tissues, is regulated by the BRB. These selective processes are achieved through either nonspecific or passive and selective energy, dependent transport mechanisms [4–7].

Several approaches have been used to study VH aiming to characterize its structural macromolecules and supramolecular organization, to identify any modification regarding metabolites composition under different conditions such as diabetes, aging, and retinal diseases, and to identify species variation in small molecule components of animal vitreous [8–12]. Recently, a ^1^H NMR-based topographical analysis of the VH proposed the existence of different functional areas within the VH, characterized by a different water-soluble low molecular weight metabolite composition. This pool comprised free amino acids, organic acids, osmolytes (such as* myo*-inositol and betaine), glucose, lactate, ascorbate, and others [13].

VH is a biofluid of interest in several fields including ophthalmology, internal medicine, and forensic pathology. In this latter scenario, VH is simple to collect, is isolated, is anatomically well protected, and so is less subject to exogenous contamination and putrefaction. Its chemical changes seem to occur at a slower rate compared to the other biofluids expanding its applicability on a wider range of time since death [14].

The aim of the present work was to study, by a metabolomic approach, the modifications of metabolite profile of goat VH samples obtained from time-related sequential withdrawals after death. Samples were collected and analyzed by ^1^H NMR spectroscopy for the simultaneous determination of all the detectable low molecular weight metabolites. Spectral data were analyzed by Principal Component Analysis (PCA) and Orthogonal Projection to Latent Structures (OPLS) regression technique. Multivariate statistical data analysis (MVA), as opposed to univariate tests, takes into consideration the correlations among the variables and all the metabolite differences simultaneously, thus giving a wider picture of modifications undergoing inside the analyzed system.

## 2. Materials and Methods

### 2.1. Collection and Preparation of Samples

Goat heads of young adult individuals, that passed the standard controls for food consumption, were obtained after animal sacrifice, by means of jugular vein incision on an electrically stunned animal, from a local slaughterhouse (CO.AL.BE. dei F.lli Contu & C. s.n.c., Selargius, Cagliari, Italy). Goat heads represent waste material, so there was neither need of an* ad hoc* animal protocol nor associated costs. Heads were stored in the morgue of the Forensic Science Unit (University of Cagliari, Italy) and kept at constant room temperature (*T* = 23°C) until the samples collection was achieved.

Collection of approximately 1 mL of VH samples was done using a 5 mL syringe G22 through a scleral puncture, at the lateral canthus from intact heads, at different times: immediately after death (t0, *n* = 3), and after 6 (t6, *n* = 3), 12 (t12, *n* = 4), 18 (t18, *n* = 5), and 24 (t24, *n* = 5) hours. 10 *μ*L of a 10% w/w aqueous solution of sodium azide (NaN_3_) was added to each sample in order to avoid bacterial growth. A single sample of VH was withdrawn from each eye. VH samples taken from eyes of the same head were collected at different times. Once collected, VH samples were immediately frozen at −80°C. The ^1^H NMR analysis was performed within three months after collection. Before the NMR analysis, samples were thawed and filtered using a 30 kDa filter unit (Amicon-30 kDa; Merck Millipore, Darmstadt, Germany) in order to remove macromolecules and active enzymes. Filters were previously washed out from glycerol by adding 500 *μ*L of distilled water and by centrifuging for 10 min at 10000 rpm at room temperature for 15 times. For the NMR analysis, 250 *μ*L of filtered vitreous sample was diluted with 350 *μ*L of a 0.33 M phosphate buffer solution (pH = 7.4) in D_2_O (99,9%, Cambridge Isotope Laboratories Inc., Andover, USA) containing the internal standard sodium 3-(trimethylsilyl)propionate-2,2,3,3,-*d*_4_ (TSP, 98 atom % D, Sigma-Aldrich, Milan) at a 0.75 mM final concentration and transferred into a 5 mm NMR tube.

### 2.2. ^1^H NMR Experiments

^1^H NMR experiments were carried out on a Varian UNITY INOVA 500 spectrometer (Agilent Technologies, CA, USA) operating at 499.839 MHz. Spectra were acquired at 300 K using the standard 1D-NOESY pulse sequence for water suppression with a mixing time of 1 ms and a recycle time of 21.5 s. Spectra were recorded with a spectral width of 6000 Hz, a 90° pulse, and 128 scans. Prior to Fourier transformation the free induction decays (FID) were multiplied by an exponential weighting function equivalent to a line broadening of 0.5 Hz and zero-filled to 64 K. All spectra were phased and baseline corrected using MestReNova software (Version 9.0, Mestrelab Research S.L.). Chemical shifts were referred to the TSP single resonance at 0.00 ppm. 2D NMR ^1^H-^1^H COSY spectra were acquired with a spectral width of 6000 Hz in both dimensions, 4096 data points and 512 increments with 64 transients per increment.

### 2.3. Data Pretreatment and Multivariate Statistical Data Analysis

The ^1^H NMR spectral region 0.80–9.00 ppm was segmented into regions (bins) of 0.02 ppm width (bucketing procedure). The region between 4.66 and 5.18 ppm was excluded from the analysis as it shows artifacts arising from water signal suppression. A total of 384 bins were obtained. The integrated area within each bin was normalized to a constant sum of 100 for each spectrum in order to minimize the effects of variable concentration among different samples. The final data set (**X** matrix) was imported into the SIMCA-P+ program (Version 13.0, Umetrics, Sweden), mean-centered and Pareto scaled column wise. In this work, Principal Component Analysis (PCA) and Orthogonal Projection to Latent Structures (OPLS) regression [15] modeling were performed. PLS-1 algorithm was used, with time as reference value and results interpreted based on the loading S-plot [16]. The S-plot visualizes both covariance and correlation structure between the variables and time. In this plot, magnitude (modelled covariation) and reliability (modelled correlation) are visualized in the *x*-axis and *y*-axis, respectively. High reliability means high effect and lower uncertainty for putative biomarker. In ^1^H NMR spectroscopic data the peak magnitude is important as peaks with low magnitude are close to the noise level and thus have a higher risk for spurious correlation. To overcome this bias Pareto scaling is suggested as method of choice. On the other hand, this scaling procedure tends to underestimate small signals that can contain altogether precious information [17]. By the use of the S-plot, taking into account model correlations, it is possible to investigate the reliability of small signals not taken into account by the model covariance under Pareto scaling. OPLS model quality was evaluated based on the parameters *R* ^2^ *Y* (goodness of fitting, i.e., fraction of *Y* variation modelled by the predictive component) and *Q* ^2^ *Y* (goodness of prediction) as determined through the default leave-1/7th-out cross validation test.

## 3. Results

**Figure 1:**
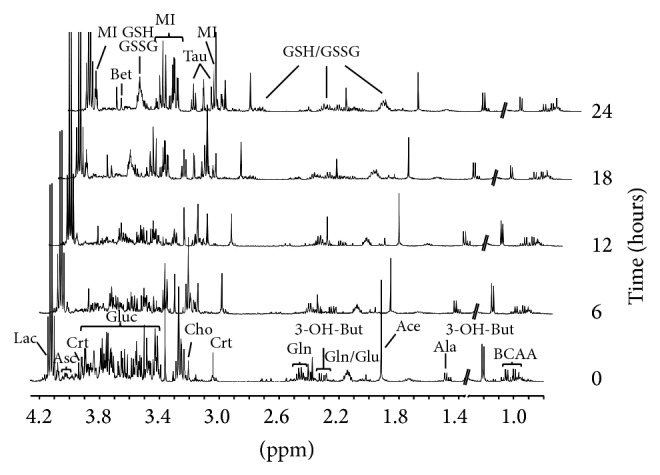
Aliphatic region (0.8–4.3 ppm) of the ^1^H NMR spectra of VH samples withdrawn at different times after death (0 and 6, 12, 18, and 24 hours). The region 1.30–1.36 ppm, due to the disproportionate intensity of the lactate doublet, is not shown. Major assignments are reported. Branched chain amino acids (BCAA: isoleucine, leucine, and valine), 3-hydroxybutyrate (3-OH-But), alanine (Ala), acetate (Ace), glutamate (Glu), glutamine (Gln), glutathione in its reduced and oxidized forms (GSH/GSSG), creatine (Crt), choline/phosphocholine (Cho), taurine (Tau), glucose (Gluc), betaine (Bet),* myo*-inositol (MI), ascorbate (Asc), and lactate (Lac).

A total of 20 VH samples, withdrawn at different times (time 0 and after 6, 12, 18, and 24 hours) after death, were analyzed. The ^1^H NMR spectra of the VH filtrates showed a hundred of resonances arising from the different functional groups of the low molecular weight water-soluble components, giving a unique sample metabolite profile.  Figure 1 shows the aliphatic region of representative ^1^H NMR spectra, with major assignments, of the VH samples for each time point. Assignments were based on literature data [12, 13], on 2D NMR experiments, on the Chenomx NMR suite 7.1 database (Chenomx Inc., Edmonton, Alberta, Canada), and by the use of standard compounds. Among the identified metabolites, there were amino acids, organic acids, sugars, nucleotides and derivatives, and others. By a visual comparative analysis of the spectra, it is possible to see, over time, gradual spectral changes of the overall profile with consistent modifications in specific spectral regions.

**Figure 2:**
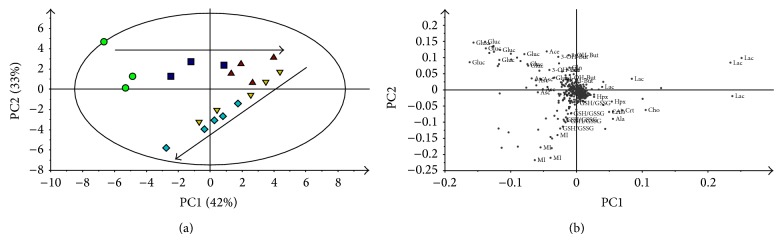
PCA applied to the ^1^H NMR spectral data of VH samples withdrawn at different times after death (0 and 6, 12, 18, and 24 hours). (a) PC1 versus PC2 scores plot (in brackets, the explained variance). Green circles = samples at time 0; blue boxes = samples at 6 hours; red triangles = samples at 12 hours; yellow inverted triangles = samples at 18 hours; light blue diamonds = samples at 24 hours. Arrows are arbitrarily drawn and represent time-related trajectories. (b) PC1 versus PC2 loadings plot. Assignments of major contributions are given. 3-hydroxybutyrate (3-OH-But), lactate (Lac), alanine (Ala), acetate (Ace), glutathione in its reduced and oxidized forms (GSH/GSSG), creatine (Crt), choline/phosphocholine (Cho), glucose (Gluc),* myo*-inositol (MI), ascorbate (Asc), and hypoxanthine (Hpx).

Given their complexity, the spectra were subjected to the bucketing procedure, and the resulting data were processed using MVA techniques [18]. At first, a PCA was performed for sample distribution overview. In the PCA score plot (Figure 2(a)) of the first two principal components that explained a large variance (*R* ^2^ *X* = 0.75, *Q* ^2^ *X* = 0.54), a time-related trajectory with two-step behaviour (from 0 to 12 and from 12 to 24 hours) is visible. The corresponding loading plot is shown in Figure 2(b). Here, for those variables (bins) more important in sample distribution, assigned metabolites are reported. From the analysis of Figure 2(b), we can see that glucose characterizes t0 samples; lactate describes the multivariate space where samples t12 and t18 lie, and hypoxanthine,* myo*-inositol, and total glutathione (in its reduced, GSH, and oxidized, GSSG, forms) describe the region of t18 and t24 samples. Other metabolites, such as choline/phosphocholine, creatine, and alanine, being in correspondence of the time-related trajectories, have also a role in sample distribution.

**Figure 3:**
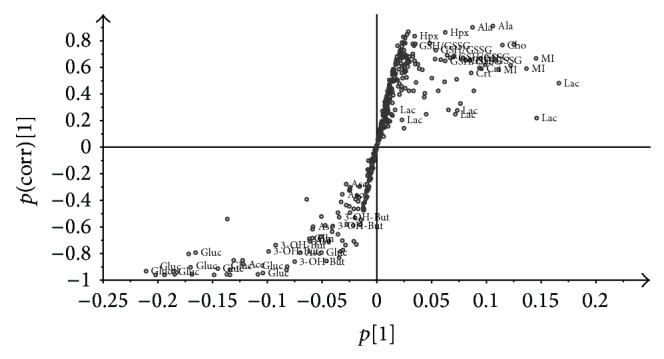
Loading S-plot of OPLS applied to the ^1^H NMR spectral data of VH samples withdrawn at different times after death (0 and 6, 12, 18, and 24 hours); *x*-axis = loadings magnitude and *y*-axis = loadings reliability. Assignments of major contributions are given. 3-hydroxybutyrate (3-OH-But), lactate (Lac), alanine (Ala), acetate (Ace), glutathione in its reduced and oxidized forms (GSH/GSSG), creatine (Crt), choline/phosphocholine (Cho), glucose (Gluc),* myo*-inositol (MI), ascorbate (Asc), and hypoxanthine (Hpx).

To ascertain whether spectral data modifications correlate with time we performed an OPLS regression. It resulted in a two-component model (one predictive and one orthogonal) with *R* ^2^ *Y* = 0.90 and *Q* ^2^ *Y* = 0.79 that indicate an excellent performance. A clear visualization of the most correlated data can be obtained by the S-plot reported in Figure 3. Here, in the *x*-axis we report magnitude of loadings (modelled covariation) and in the *y*-axis their reliability (modelled correlation). In the upper side of the S-shape we identified hypoxanthine and alanine strongly positively correlated with time, together with choline/phosphocholine,* myo*-inositol, creatine, and total glutathione. In the opposite side of the S-shape, that is, strongly inversely correlated with time, we identified glucose and 3-hydroxybutyrate.

**Figure 4:**
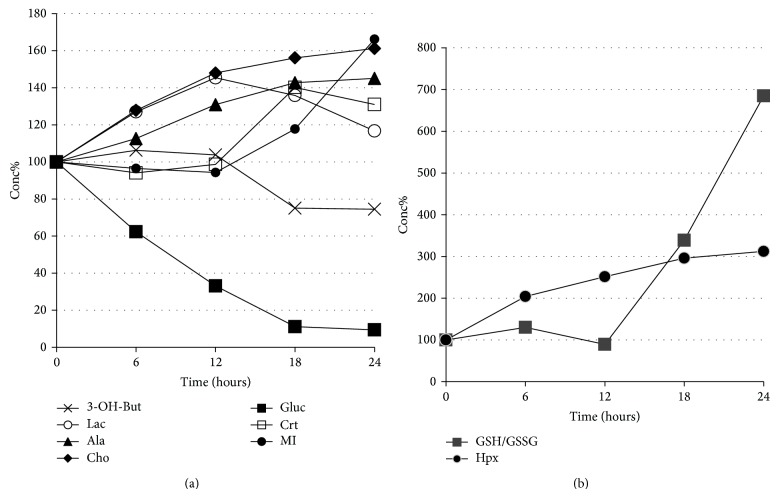
Concentration %, with respect to the t0 samples, versus time of (a) glucose (Gluc),* myo*-inositol (MI), creatine (Crt), alanine (Ala), 3-hydroxybutyrate (3-OH-But), choline/phosphocholine (Cho), and lactate (Lac); (b) glutathione, in its reduced and oxidized forms (GSH/GSSG), and hypoxanthine (Hpx). Chemical shift range (ppm) of the corresponding integrated areas in the ^1^H NMR spectra: Gluc 3.46–3.48, MI 4.06–4.08, Crt 3.92–3.94, Ala 1.46–1.48, 3-OH-But 1.20–1.22, Cho 3.20–3.22, Lac 1.34–1.36, GSH/GSSG 2.96–2.98, and Hpx 8.20–8.22.

In order to study the time-related variation of each one of these metabolites we performed a semiquantitative analysis of the corresponding spectral data. Bins where metabolites resonate with minimal overlapping were chosen. The spectral integrated areas of the selected bins were collected and reported in Table 1 as mean and standard deviation values for each metabolite at each time point. Table 1 also reports the statistically significant Student's* t*-test *P* values for the different time steps. It can be seen that, even if the associated errors are sometimes large, the global trend of metabolite variation is significant. In particular, the *P* values indicate significant different mean values going from 0 to 24 hours for all the examined metabolites. The results are graphically reported in Figure 4, where the mean values for each time point were normalized to the t0 mean value set as 100. Data concerning hypoxanthine and total glutathione that showed minimal concentrations in samples t0 are reported in Figure 4(b). The overall trend of data in Figure 4 confirms our interpretation of PCA results, where at 12 hours a different behaviour of several metabolite relative concentrations over time can be observed.

## 4. Discussion

In this study, based on a ^1^H NMR multivariate approach, variations in VH metabolite profile in time-related sequential sampling were monitored in an animal model. We followed a typical metabolomic workflow, including sample preparation, analysis, data processing, and data analysis. The power of this approach lies on the acquisition of analytical data in which metabolites in a cellular/biological system are identified and on the extraction of the most meaningful elements of these data using various MVA tools. Among these, unsupervised and/or supervised approaches may be applied. The former are usually applied to get an overview of sample characteristics, to detect outliers and irregularities in the data, with the PCA being the gold standard of these techniques. Among the latter, there are regression techniques that use preliminarily gathered information (measured variable), to build models, based on subtle differences between similar samples, that seek regularities in metabolite changes linked to the measured property under study. In this work, to exploit the time-related information content of the NMR data acquired on VH sampled at different time points after death, the orthogonal variation of the partial least squares (OPLS) regression analysis was used. It finds the best correlations between the NMR spectral features and time. More technically, OPLS is a regression technique that correlates** X** matrix (spectral data) with a continuous response** y** (measured time) trying to find the regularities in the** X** data that better correlate with** y**. The whole NMR spectra are used in the modelling; therefore the multivariate OPLS model results in a more comprehensive/complete point of view/picture compared to uni/bivariate approaches.

The different spectral metabolite profiles observed in this study can be interpreted as the result of the time-related VH perturbations. Metabolites most involved were glucose and 3-OH-butyrate which decrease with time and lactate, hypoxanthine, alanine, total glutathione, choline, creatine,* myo*-inositol, which increase with time.

From Figures 2(a) and 4, a marked modification of the time-related trends for several metabolites starting from the 12th hour after death can be evinced. This behavior can be tentatively explained by the energetic failure of the VH system in this crucial moment. For example, glucose, lactate, and 3-hydroxybutyrate compounds are all involved in energy metabolism. It is well known that during energy depletion, glycogen and triglyceride stores are liberated and oxidized to produce energy in the form of ATP. Under anaerobic conditions, such as in hypoxia/anoxia, an inhibition of aerobic energy metabolism and a switch towards anaerobic glycolysis occur. The time-dependent lactate rise, together with the progressive consumption of glucose, may be considered the extreme attempt of the corpse to adequately self-guarantee ATP levels even after cardiorespiratory failure. We also observed that 3-hydroxybutyrate was inversely correlated with time, with a drop after 12 hours. 3-hydroxybutyrate, normally present in the blood, is one of the ketone bodies produced in the liver, mainly from the oxidation of fatty acids, and exported to peripheral tissues to be used as an alternative energy source, especially for brain. It is possible to speculate that its behavior in our samples is determined by its progressive consumption in an oxygen-depleting environment in an attempt to provide an energetic source alternative to glucose. A theoretical bioenergetics study suggested that 3-hydroxybutyrate may be able to provide a more efficient aerobic energy source, yielding greater production of ATP* per* molecule than glucose [19].

As already reported by Alm et al., [20–22] the active transport of glucose, lactate, amino acids, and other selected molecules from the blood to the retina across the BRB takes place* via* carrier-mediated processes. It is known that the energetic failure induces loss of the functional integrity of the inner BRB [23] thus allowing uncontrolled molecular crossing of the barrier. This can be the case of MI involved in osmoregulation [24, 25], which after 12 hours showed an increase that can be explained by passive diffusion—through a concentration gradient determined by the uneven molecular distribution at the retinal-vitreous interface—into the VH chamber. If we consider that a loss of BRB functionality can have taken place, also total glutathione, found in very small quantities in the VH at t0, may leak from the surrounding tissue into the VH cavity. GSH is found in the retina, precisely in the Müller cells, where it is stored in large amounts in order to protect retinal neurons from oxidative stress. GSH supply in retina shows a decrease with aging and during certain pathological conditions such as diabetic retinopathy [26, 27]. Total glutathione trend in VH samples is shown in Figure 4(b). Among the other identified and measured metabolites we found creatine and choline, both increasing with time. The former is a guanidino compound synthesized from the amino acids arginine, glycine, and methionine. Its main function, although additional roles—such as direct scavenging of reactive oxygen species (ROS)—are under investigation, is to restore ATP from ADP, acting as a catalyzer. Creatine was also shown to have an important role in osmoregulation as well as glycerol [28]. Choline is an essential nutrient. Its role in the body is complex. It is needed for neurotransmitter synthesis (acetylcholine), cell-membrane signaling (phospholipids), lipid transport (lipoproteins), and methyl group metabolism (homocysteine reduction). Another metabolite is alanine. This amino acid has been indicated as biomarker in brain of apoptosis and/or cellular stress. Increase of alanine has been observed in meningioma and following ischemia [29]. In the present work, we found that hypoxanthine is one of the metabolites directly correlated with time; its trend with respect to time is reported in Figure 4(b). It is worth mentioning that, together with potassium, this compound is one of the variables used in the forensic VH investigation of* post-mortem* interval. Hypoxanthine is a degradation product of adenine nucleotide metabolism. It is formed by the action of several enzymatic reactions and then diffuses along with the concentration gradient [30].

The observed increase of molecules which are well represented in the retina, such as* myo*-inositol [24] and total glutathione [6], starting from 12 h after death, suggests the progressive decline of the ATP dependent functions, together with the failure of the blood-retinal and retinal-vitreal barriers. Together with the loss of the active transport mechanism, after death, it is presumable that the eyeball, in particular the VH, achieves homeostasis both internally and with adjacent structures such as retina.

Most of the aforementioned metabolites, differently involved in the time-related modification of goat VH composition, were recently described in a human population. Reinke et al. [29] investigated by a ^1^H NMR approach a significant population of neonates born, after a clinically documented neonatal asphyxia, with or without developing hypoxic ischemic encephalopathy (HIE). Among the metabolites peculiar to neonatal asphyxia and to HIE, the authors reported alanine, choline, creatine, lactate,* myo*-inositol, glucose, and 3-hydroxybutyrate. These results strengthen the hypothesis that the ongoing metabolomic profile, described in our animal model, may be representative of the energy metabolism derangement secondary to death and may be applied to human samples.

## 5. Conclusions 

The present study, using a ^1^H NMR multivariate analysis of goat VH samples, showed a clear time-related change of the metabolic profile, which involves mainly central energy, amino acids, and ketones metabolisms.

This approach proved to be able to describe the modifications occurring—in a time-related means after death—in a well preserved biofluid, such as VH, by the analysis of a global metabolite profile rather than one or few metabolites. Further studies have to be carried out to test the feasibility of this approach to a human forensic* post-mortem* interval analysis.

## Figures and Tables

**Table 1:** Mean and standard deviation (SD) values of the spectral integrated area^a^ corresponding to the identified metabolites at each time point, with statistically significant Student's *t*-test *P* values^b^.

Metabolite	t0		t6			t12				t18				t24			
	Mean	SD	Mean	SD	*P*t0^c^	Mean	SD	*P*t6^d^	*P*t0^c^	Mean	SD	*P*t12^e^	*P*t0^c^	Mean	SD	*P*t18^f^	*P*t0^c^
3-OH-But	0.7	0.1	0.71	0.06		0.7	0.1			0.5	0.1	∗∗	∗	0.5	0.06		∗
Lac	20.6	0.8	26	2	∗∗	30	2	∗	∗∗∗	28	3		∗∗	24	2	∗∗	∗∗∗
Ala	0.4	0.1	0.42	0.02		0.49	0.03	∗∗		0.54	0.02	∗∗		0.54	0.03		∗
GSH/GSSG	0.03	0.03	0.03	0.03		0.02	0.02		∗	0.09	0.09			0.18	0.09	∗	∗∗
Cho	0.7	0.1	0.9	0.1	∗	1.04	0.01			1.1	0.2		∗∗	1.13	0.08		∗∗
Gluc	0.8	0.3	0.52	0.05		0.27	0.07	∗∗∗		0.09	0.02	∗∗	∗	0.078	0.008	∗	∗
Crt	0.7	0.2	0.6	0.1		0.66	0.05			0.9	0.2	∗	∗	0.9	0.1		∗
MI	1.0	0.1	0.9	0.2		0.9	0.1			1.1	0.4			1.6	0.4	∗	∗∗
Hpx	0.05	0.02	0.111	0.004	∗∗	0.137	0.004	∗∗∗	∗∗∗	0.16	0.03	∗	∗∗∗	0.17	0.01		∗∗∗

^a^Chemical shift range (ppm) of the corresponding integrated areas in the ^1^H NMR spectra: Gluc 3.46–3.48, MI 4.06–4.08, Crt 3.92–3.94, Ala 1.46–1.48, 3-OH-But 1.20–1.22, Cho 3.20–3.22, Lac 1.34–1.36, GSH/GSSG 2.96–2.98, and Hpx 8.20–8.22; ^b^ *P *≤ 0.01^***^, 0.01 < *P* ≤ 0.05^**^, 0.05 < *P* ≤ 0.2^*^; ^c^ *P* values versus t0; ^d^ *P* values versus t6; ^e^ *P* values versus t12; ^f^ *P* values versus t18.
